# Efficacy of Clindamycin in Preventing Abortion and Vertical Transmission of *Toxoplasma gondii *(PRU Strain) Infection in Pregnant BALB/c Mice

**DOI:** 10.5812/ijpr-150424

**Published:** 2024-10-14

**Authors:** Mitra Sadeghi, Seyed Abdollah Hosseini, Shahabeddin Sarvi, Pedram Ebrahimnejad, Hossein Asgarian Omran, Zohreh Zare, Shirzad Gholami, Alireza Khalilian, Mostafa Tork, Ahmad Daryani, Sargis A Aghayan

**Affiliations:** 1 Toxoplasmosis Research Center, Communicable Disease Institute, Mazandaran University of Medical Sciences, Sari, Iran; 2 Student Research Committee, Mazandaran University of Medical Sciences, Sari, Iran; 3 Department of Parasitology and Mycology, Faculty of Medicine, Mazandaran University of Medical Science, Sari, Iran; 4 Department of Medicinal, Chemistry and Pharmaceutical Sciences Research Center, Faculty of Pharmacy, Mazandaran University of Medical Sciences, Sari, Iran; 5 Department of Immunology, School of Medicine, Mazandaran University of Medical Sciences, Sari, Iran; 6 Department of Anatomical Sciences, Molecular and Cell Biology Research Center, Faculty of Medicine, Mazandaran University of Medical Sciences, Sari, Iran; 7 Department of Biostatistics, Mazandaran University of Medical Sciences, Sari, Iran; 8 Laboratory of Molecular Parasitology, Scientific Center of Zoology and Hydroecology, Yerevan, Armenia; 9 Laboratory of Zoology, Research Institute of Biology, Yerevan State University, Yerevan, Armenia

**Keywords:** *Toxoplasma gondii*, Pru Strain, Congenital, Clindamycin, *In Vivo*

## Abstract

**Background:**

*Toxoplasma gondii *transmission can occur during pregnancy if the mother contracts the infection for the first time. Treatment strategies include the use of antimicrobial medications and providing supportive care. Spiramycin is commonly used to treat toxoplasmosis in pregnant women and to hinder the disease's transmission. However, its ability to treat the fetus is questionable due to its limited capacity to cross the placental barrier. Additionally, economic constraints and sanctions may impede access to this medication.

**Objectives:**

Consequently, in search of an effective treatment, for the first time in Iran, the effectiveness of clindamycin in preventing abortion and vertical transmission of the PRU strain of *T. gondii* infection in pregnant mice was evaluated.

**Methods:**

On the twelfth day of gestation, pregnant mice were exposed to *T. gondii* and subsequently received treatment with either clindamycin or spiramycin. This resulted in the establishment of four distinct groups: A normal control group, an infected group without treatment, an infected group treated with clindamycin, and another infected group treated with spiramycin. Following these interventions, a series of parasitological evaluations (including microscopic examination and real-time PCR), histopathological evaluations, and immunological assessments were conducted.

**Results:**

The findings showed a significant reduction in the number of cysts in the eye and brain (ranging from 77.32% to 90.72%) among the groups treated with clindamycin and spiramycin compared to the control group. Furthermore, treatment with clindamycin, like treatment with spiramycin, was able to suppress inflammatory changes, prevent cell death, and reduce vascular cuffs in the brain, as well as decrease bleeding, placental thrombosis, and the accumulation of inflammatory cells in the placenta. Clindamycin was also effective in diminishing retinal folds, tiny retinal bleeds, and cell vacuolation in eye tissues. Immunologically, treatment in both the spiramycin and clindamycin groups resulted in a decrease in the level of the cytokine TNF-α, indicating an increase in the cellular immune response. In addition, increased levels of IL-10 in the treated infected groups could contribute to the reduction of TNF-α production.

**Conclusions:**

Typically, spiramycin is the first choice for treating congenital toxoplasmosis, but clindamycin can be a useful substitute or additional treatment when resistance to primary medications occurs, when there is intolerance, or when access to the main drugs is restricted.

## 1. Background

Toxoplasmosis is caused by an obligate intracellular protozoan known as *Toxoplasma gondii *(*T. gondii*), capable of infecting a wide range of warm-blooded vertebrates (1). Humans can contract this infection by consuming the cyst form present in undercooked or raw meat or the oocyst form through exposure to cat feces, contaminated food, or soil. Transmission can also occur congenitally. Although infection in humans typically shows no symptoms, it can lead to serious complications in individuals with compromised immune systems, particularly in those with AIDS/HIV+, as well as in infants and pregnant women (2).

Congenital toxoplasmosis stands out as a notable complication arising from toxoplasmosis infection, with an estimated global occurrence of 1 to 6 cases per 1,000 births (3). Three primary genotypes of *T. gondii*, labeled I, II, and III, have been identified. In Europe and the USA, Type II is linked to approximately 80% of congenital infections, which can range from subclinical to severe and potentially fatal cases. Type I is commonly associated with the more severe manifestations of the disease (4).

A congenital infection can occur if a woman contracts *T. gondii* for the first time during pregnancy (3). If the mother experiences an acute infection, *T. gondii* can be transmitted hematogenously across the placenta and into the fetal bloodstream. The timing during pregnancy when the mother is exposed to *T. gondii*, along with the stage of gestation, greatly influences the risk of infection and potential outcomes for the embryo or fetus (5).

Babies born to mothers who contract an infection during the first or second trimester of pregnancy often exhibit severe signs of congenital toxoplasmosis, including brain calcifications, brain abscesses, hydrocephalus, microcephaly, retinochoroiditis, and ascites. In some cases, the infection can lead to miscarriage or stillbirth (6). The impact of antiparasitic therapy on mother-to-child transmission is a subject of ongoing debate. Nonetheless, it is advised to initiate treatment promptly upon diagnosis. Typically, toxoplasmosis is managed with pyrimethamine and sulfadiazine. However, during pregnancy, particularly within the first 18 weeks, pyrimethamine-sulfadiazine treatment may result in teratogenic effects and potential miscarriage. Consequently, spiramycin is the preferred medication to prevent infection (7). While spiramycin, a macrolide antibiotic, is adept at preventing parasite transmission and lessening the severity of fetal infection (8), its effectiveness in reaching fetal tissues is limited. Prenatal administration can decrease transmission risk by up to 60% (9). Nonetheless, economic constraints and sanctions in certain regions can restrict access to spiramycin. As a result, alternative medications such as clindamycin and azithromycin are under investigation as possible treatments.

Clindamycin, a *lincosamide* antibiotic akin to macrolides, has the ability to permeate the placental barrier during pregnancy, achieving umbilical cord serum levels approximately half that of maternal serum. Substantial evidence indicates that clindamycin is safe for use in pregnant women, with no teratogenic effects observed following topical or oral administration (10, 11). Nonetheless, experimental studies examining clindamycin’s efficacy in treating congenital toxoplasmosis during pregnancy are sparse. Considering the risk of *T. gondii*’s congenital transmission and its enduring presence, additional research is essential to assess clindamycin’s effectiveness in preventing transmission and its influence on the infection’s persistence.

## 2. Objectives

Hence, this research was undertaken for the first time in Iran to explore the potential of clindamycin in either preventing or diminishing the vertical transmission of toxoplasmosis infection, specifically caused by the Type II (PRU strain) of *T. gondii*, utilizing a murine model.

## 3. Methods

### 3.1. Bioethics

This project, approved by the Ethics Committee of Mazandaran University of Medical Sciences (IR.MAZUMS.AEC.1402.010), followed institutional guidelines for animal experimentation. Male and female BALB/c mice aged 10 - 12 weeks and weighing 30 ± 2 g were used and maintained under specific conditions.

### 3.2. Parasite

Cysts from the PRU strain of *T. gondii* were sourced from the Toxoplasmosis Research Center (TRC) at Mazandaran University of Medical Sciences, Sari, Iran. To enhance the virulence of the parasites, they were propagated through intraperitoneal inoculation in BALB/c mice. Two months later, the brains of the infected mice were extracted, homogenized in PBS, and used for subsequent injections.

### 3.3. Drugs Treatment

(1) Clindamycin hydrochloride hydrate, provided in powdered form by Alborz Company based in Tehran, Iran, was administered orally via gavage at a dosage of 200 mg/kg body weight daily.

(2) Spiramycin was administered in the form of 3 M.I.U tablets, which were crushed into a powder. This powder was then dissolved in water and orally delivered to the mice at a dosage of 400 mg/kg body weight daily.

### 3.4. Mating and Infection

Mating of female mice with males was conducted in a regulated environment. The onset of pregnancy was verified through observed weight gain and the presence of a vaginal plug. Subsequently, the males were separated, and groups of three females were housed together until the time of infection (12). On the twelfth day of gestation, the mice received an intraperitoneal injection containing 20 cysts of the *T. gondii* PRU strain suspended in PBS in preparation for subsequent analysis.

### 3.5. Experimental Groups

In the conducted experiment, 24 pregnant mice were allocated into four distinct groups: Group 1 served as the normal group, being uninfected and untreated; group 2 was the negative control, infected but not treated; group 3 was infected with *T. gondii* and received clindamycin treatment at a dose of 200 mg/kg; and group 4, the positive control, was infected and treated with spiramycin at a dose of 400 mg/kg. Both groups 3 and 4 were administered the respective drugs orally from the time of parasite inoculation until delivery, with dosages aligned with those recommended for pregnant women.

### 3.6. Tissue Sampling

#### 3.6.1. Placental Tissue Collection and Analysis

Two days before the expected delivery date, pregnant BALB/c mice underwent a cesarean section to surgically remove their placentas. The collected placental tissues were then analyzed for the presence of *T. gondii* parasites using the following methods.

#### 3.6.2. Parasite Load Evaluation

A 10 μL sample of the homogenized placental tissue was examined under a microscope to assess the parasite load. The remaining homogenized tissue from the left halves was preserved at -20°C for further evaluation of parasite load using real-time PCR.

#### 3.6.3. Histopathological Examination

The right halves of the placental tissues were preserved in 10% formalin for subsequent histopathological examination.

#### 3.6.4. Offspring Tissue Collection and Analysis

Six weeks after delivery, the offspring mice were humanely euthanized, and their brain and eye tissues were collected. The collected tissues were then processed as follows: The tissues were bisected into left and right sections; the left halves were homogenized and analyzed for parasite load using the methods described above for placental tissues; the right halves were preserved in 10% formalin for histopathological examination.

This study aimed to evaluate the transmission of *T. gondii* from the placenta to the offspring in a BALB/c mouse model, using a combination of microscopic examination, real-time PCR, and histopathological analysis of the collected tissues.

### 3.7. Counting the Toxoplasma gondii Tissue Cysts Number

To assess the effects of the drug, the number of cysts present in 0.01 gram of mouse brain and eye tissues was evaluated by microscopic examination (NIKON, Japan) at 40× magnification and Giemsa staining (MERCK, Germany) (13).

### 3.8. Real-time PCR

**Figure 1.**
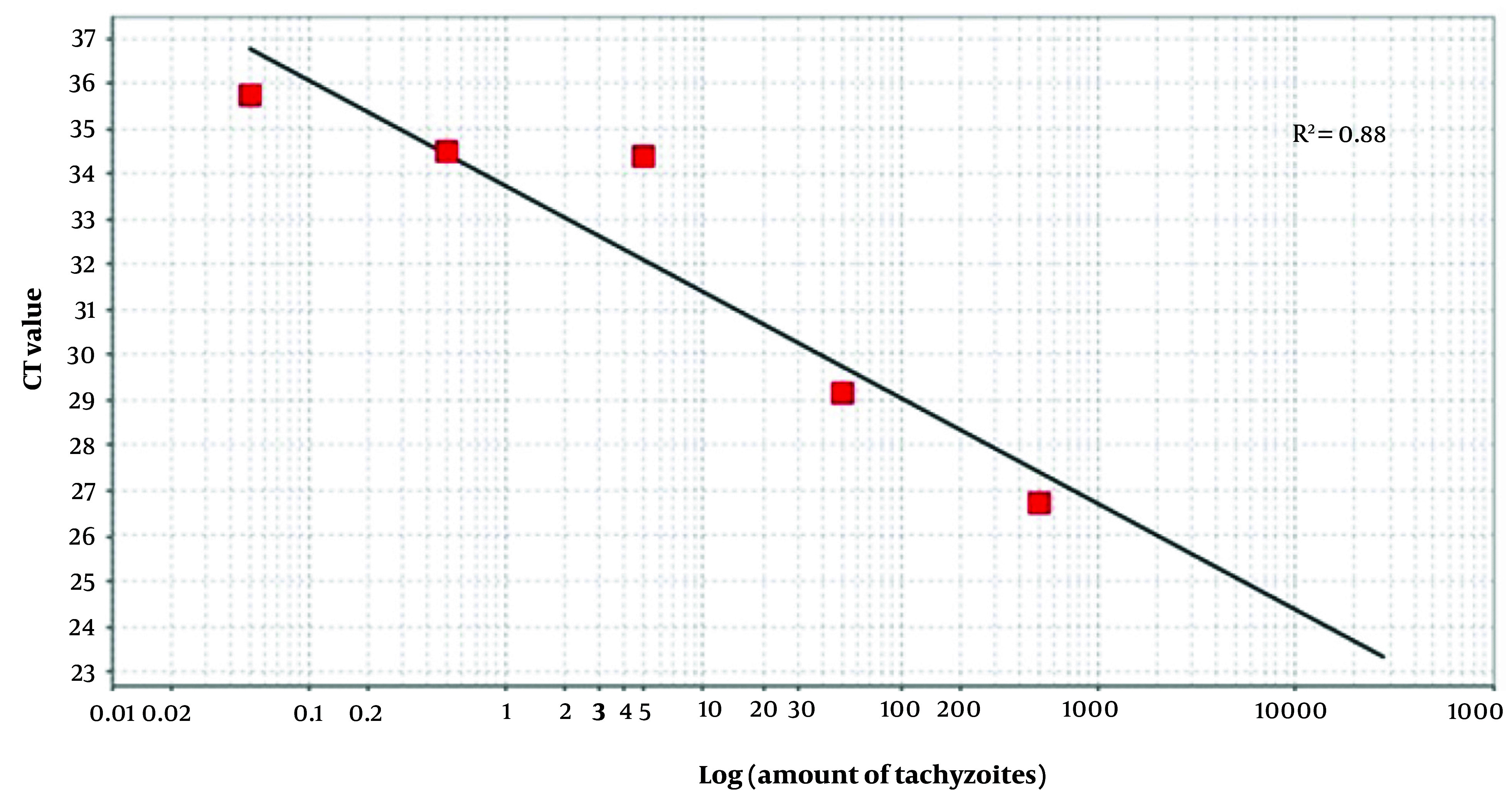
Development of a standard curve for *T. gondii* quantification. Serial dilutions of *T. gondii* DNA, from 500 to 0.05 tachyzoites, were used as templates for real-time PCR analysis and threshold cycle (CT) values were plotted against tachyzoites.

DNA was extracted from the tissues (brains and eyes of newborn mice and placentas of pregnant mice) using the animal tissue DNA isolation kit (DENAzist, Iran). The B1 gene, including forward primers 5'-CGTGTTCGTCTCCATTCC-3' and reverse primers 5'-TGCCTTCTCTTTGTTACTCTAC-3', aimed to amplify a 167 bp fragment using AB Applied Biosystems (the initial step in real-time PCR), following previously established protocols (14), to determine the *T. gondii* load. The parasite burden of *T. gondii* was assessed by comparing the threshold cycle (CT) values with standard curves derived from DNA of serially diluted RH strain tachyzoites (500 - 0.05/mL), as shown in Figure 1. Distilled water and DNA from RH strain tachyzoites were employed as negative and positive controls, respectively. 

### 3.9. Histopathological Study

Tissue specimens from each group were separately harvested and fixed in neutral buffered formalin for 48 hours. After processing for paraffin embedding, 5-μm-thick sections were prepared using a rotary microtome (Leica Model RM 2145, Germany) and stained with hematoxylin and eosin (H&E). The prepared slides were evaluated for any histopathological changes under light microscopy (NIKON, Japan) (15).

### 3.10. Immunological Study and Cytokines Assay

To measure cytokines, blood samples were obtained from the hearts of mice six weeks post-birth using sterile vacuum tubes containing EDTA. Serum levels of TNF-α and IL-10 were then quantified using an ELISA kit (Carmania Parsgen, Iran), following the guidelines provided by the manufacturer. Data were presented in pg/mL (16).

### 3.11. Statistical Analysis

The data gathered were subjected to statistical analysis using GraphPad Prism software version 8.0 (GraphPad Software, Inc., San Diego, CA, USA) and SPSS software version 26. The differences between the test groups (both positive and negative) and the control groups were evaluated using the *t*-test and Mann Whitney test. A P-value of 0.05 or less was considered statistically significant in this study.

## 4. Results

### 4.1. Estimation of the Parasite Burden by Microscopic Examination

The results of macroscopic observation showed that no abortions occurred in mice treated with clindamycin and spiramycin. Next, we examined the groups microscopically. Table 1 demonstrates that spiramycin and clindamycin treatments led to a marked decrease in the number of brain cysts (by 90.72% and 77.32%, respectively) and eye cysts (by 90.48% and 80.95%, respectively) when compared to the untreated control group. The spiramycin-treated group exhibited the fewest brain and eye cysts, showing a significant difference from the control group (P < 0.05). Although the mice treated with spiramycin had the lowest number of brain and eye cysts compared to the positive control group, the difference in cyst numbers between the clindamycin and spiramycin groups was not statistically significant (P > 0.05).

**Table 1.** Toxoplasma Cyst Burden per Hippocampus Brain and Eye Section in Various Infected Groups ^a^

No.	Groups	Number of Cysts in Brain	P-Value		P-Value		Positive Control
			Negative Control	Positive Control	Number of Cysts in Eye ^b^	Negative Control	
**1**	PBS (negative control)	19.4 ± 3.36	-	0.000	4.2 ± 3.56	-	0.03
**2**	Clindamycin	4.4 ± 2.40	0.000	0.81	0.8 ± 0.83	0.03	0.41
**3**	Spiramycin (positive control)	1.8 ± 1.46	0.000	-	0.4 ± 0.54	0.01	-
**4**	Normal group	0	0.000	0.000	0	0.000	0.000

^a^ Values are expressed as mean ± SD.

^b^ Significant with Mann-Whitney test (P-value less than 0.05).

### 4.2. Parasite Burden by Real-time PCR

In the examined samples from mice that received treatment, there was a notable decrease in the prevalence of the B1 gene compared to the untreated control group (p < 0.05). The reduction was significantly more pronounced in the spiramycin-treated mice than in those given clindamycin. In particular, mice treated with spiramycin at a dose of 400 mg/kg/day showed the lowest levels of parasites in the brain, eye, and placenta (3.9, 1.75, and 4 parasites/mL, respectively). Although the clindamycin group exhibited a substantial reduction in gene expression and parasite burden relative to the control group, it did not achieve the same efficacy as spiramycin, with a significant statistical difference between the two treatment groups (P < 0.05), as shown in Table 2. 

**Table 2.** Comparative Analysis of CT Values and the Burden of *Toxoplasma gondii* Parasites in Various Tissues (Brain, Eye, and Placenta) Across Different Groups Utilizing Real-time PCR

Groups	CT (Mean ± SD of Parasite Load)								
	Brain	Statistical Significance (P < 0.05)		Eye	Statistical Significance (P < 0.05)		Placenta	Statistical Significance (P < 0.05)	
		Negative Control	Positive Control		Negative Control	Positive Control		Negative Control	Positive Control
**PBS (negative control)**	25.21 ± 0.89 (3850 ± 450)	-	0.000	24.86 ± 0.29 (5850 ± 2 950)	-	0.000	23.99 ± 0.92 (15500 ± 10500)	-	
**Clindamycin**	29.65 ± 1.22 (42.66 ± 97.68)	0.007	0.01	29.25 ± 0.23 (48.5 ± 1.5)	0.000	0.000	28.77 ± 0.5 (135 ± 45)	0.004	0.003
***Spiramycin*** ** (positive group)**	32.7 ± 0.26 (5.9 ± 0.1)	0.000	-	33.36 ± 0.28 (1.25 ± 0.25)	0.000	-	32.91 ± 0.54 (2.5 ± 0.1)	0.007	-
**Normal group**	37.26 ± 0.1	0.002	0.005	37.39 ± 0.21	0.002	0.005	37.26 ± 0.1	0.002	0.005

### 4.3. Histopathological Evaluation

**Figure 2.**
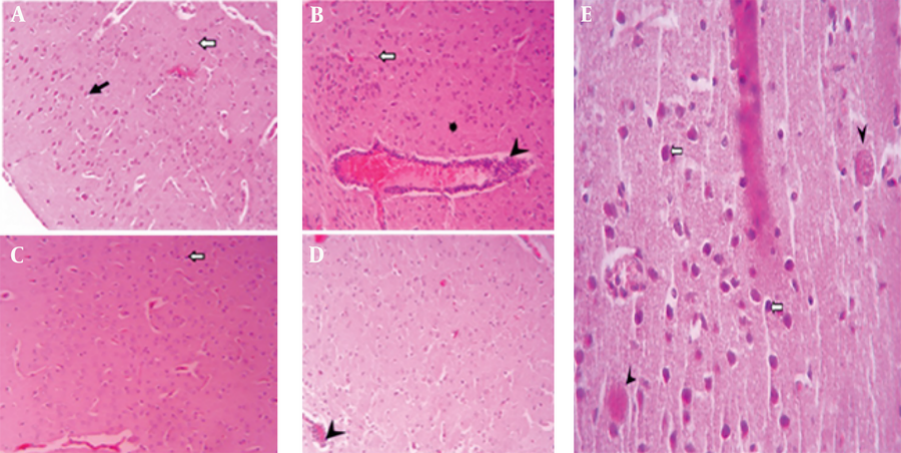
Microscopic images of mouse brain tissue, H&E staining, 200× magnification. A, the control group displays normal brain structures, including neurons (black arrow) and glial cells (white arrow); B, brain tissue from mice infected with the PRU strain and untreated; C, tissue from PRU-infected mice treated with spiramycin; D, tissue from PRU-infected mice treated with clindamycin, showing signs of inflammation such as glial cell proliferation (black arrow), vacuolation in the neuropil (star), neurons that are shrunken and darkly stained (white arrow), and perivascular cuffs (head arrow); E, a distinct cyst image (arrowhead) and pyknotic neurons (white arrows) are observed

**Figure 3.**
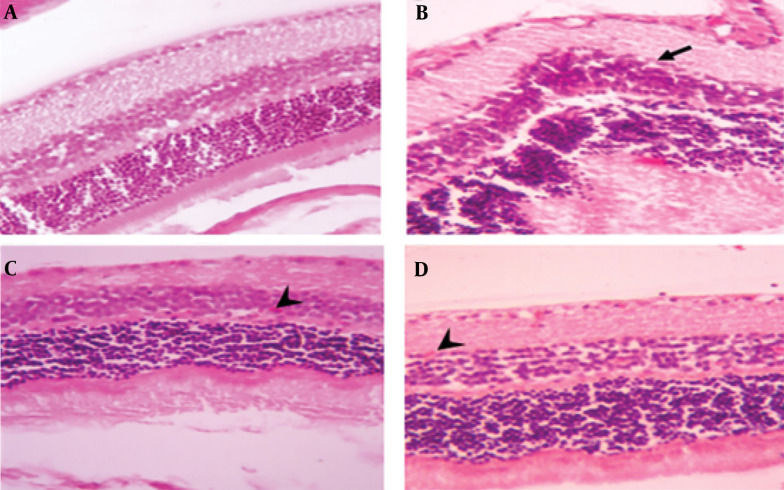
Microscopic image of mouse eye tissue, H&E staining, 400× magnification. A, the control group displays normal eye tissue layers; B, tissue sections from PRU strain-infected groups without medication; C, sections from spiramycin-treated, infected groups; D, clindamycin-treated, infected tissue, illustrating retinal folds and structural damage (indicated by black arrow) as well as retinal micro hemorrhages (denoted by arrowhead)

**Figure 4.**
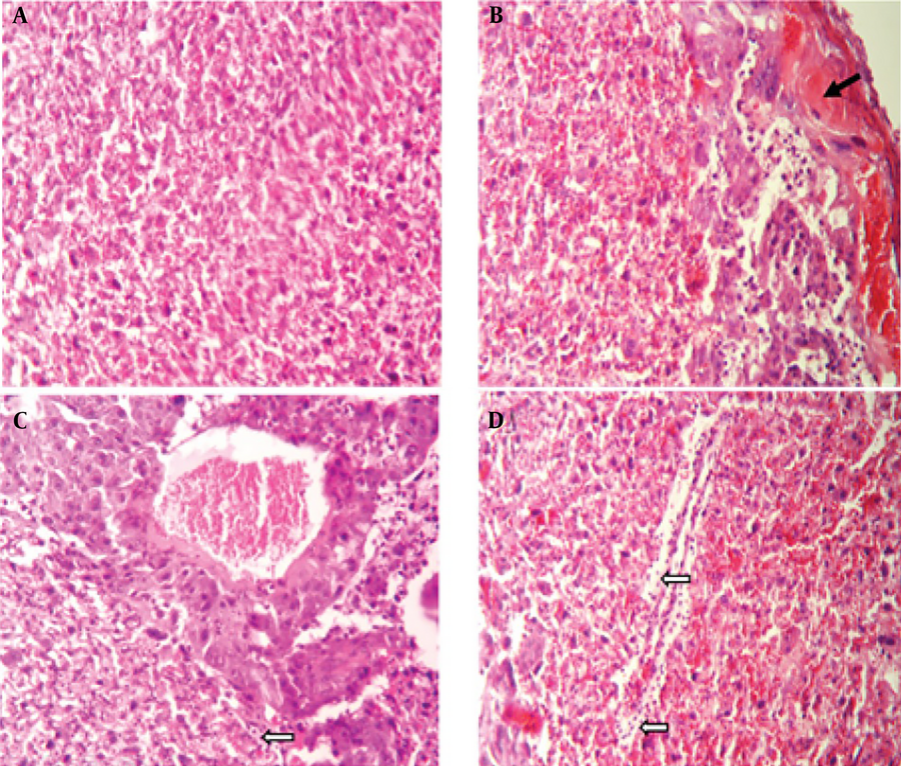
Microscopic examination of mouse placenta tissue, H&E staining, 200× magnification. A, the control group displays normal placental labyrinth layer tissue; B, histological sections of placenta tissue from groups infected with the PRU strain, without any drug treatment; C, show infection treated with spiramycin; and D, infection treated with clindamycin, demonstrate thrombosis and hemorrhage (indicated by black arrow), an accumulation of inflammatory cells (white arrows), and local calcification (head arrows)

A histopathological examination of tissues from uninfected mice revealed no abnormalities, with cells appearing normal. In contrast, brain tissue from untreated, infected mice displayed signs of inflammation, necrosis, glial cell proliferation, perivascular cuffing, and widespread neurodegeneration. However, treatment with spiramycin and clindamycin mitigated these effects. Notably, spiramycin-treated mice showed minimal neuronal death and inflammation, with an absence of perivascular cuffing (Figure 2). In the eyes of untreated infected mice, severe structural damage was observed, including retinal folding, hemorrhages, and cellular vacuolation. Both drugs improved ocular conditions compared to untreated mice, but clindamycin was notably more effective than spiramycin (Figure 3). Additionally, spiramycin significantly reduced placental damage in treated mice, evidenced by decreased hemorrhage, thrombosis, inflammatory cell accumulation in the labyrinth zone and decidua, and localized calcification in the labyrinth zone (Figure 4). 

### 4.4. Immunological Study

**Figure 5.**
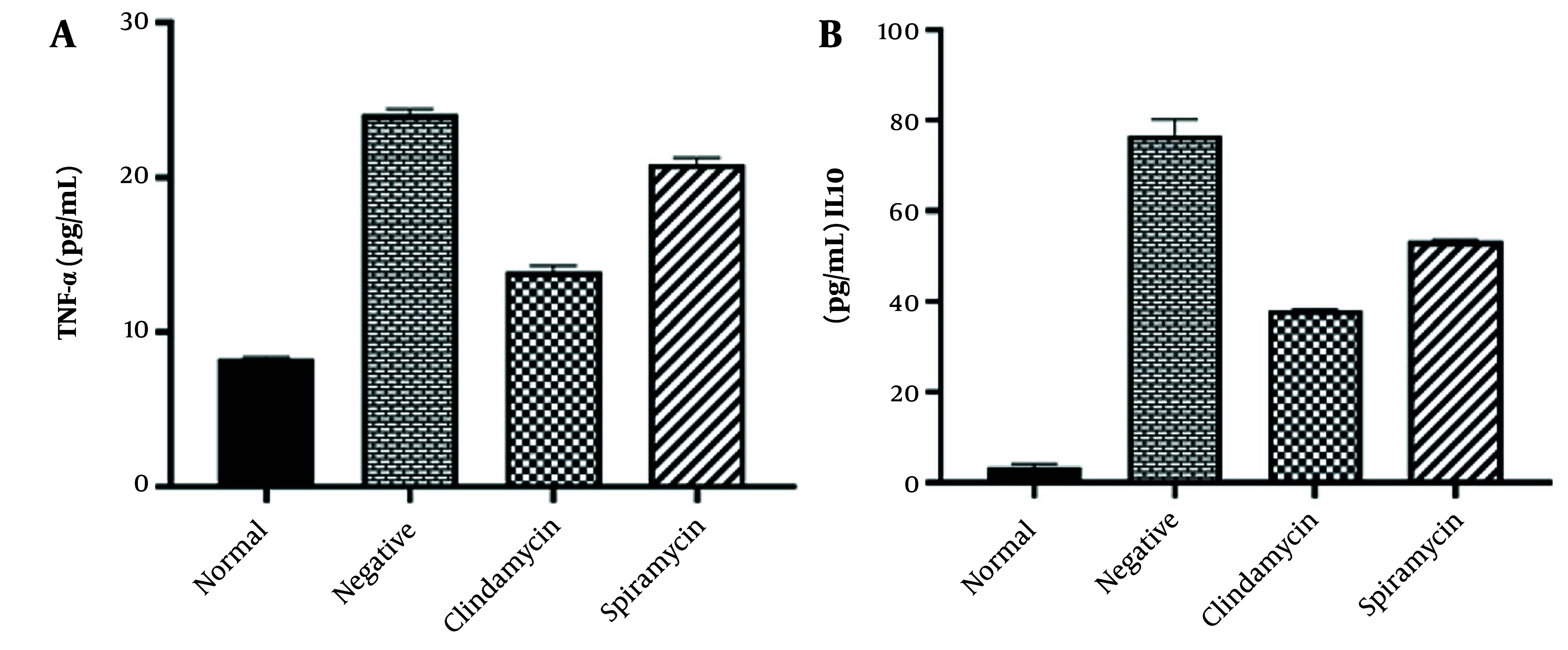
Serum Level of IL-10 and TNF-α of different groups. Data represent the mean ± standard error of the mean (SEM)

The levels of IL-10 and TNF-α were assessed in the serum of all mouse groups, with the normal group exhibiting the lowest levels of detectable serum cytokines. In contrast, the negative control group showed the highest production of IL-10 and TNF-α cytokines. Additionally, mice treated with clindamycin exhibited moderate levels of secretion of both cytokines compared to the negative control group. Compared to the spiramycin group, mice treated with clindamycin showed lower levels of IL-10 and TNF-α, and this difference was statistically significant (P < 0.05). Immunological analysis revealed significant differences in IL-10 and TNF-α levels among all groups when compared to the infected control group, as shown in Figure 5. 

## 5. Discussion

Treatment for congenital toxoplasmosis involves the use of antimicrobial medication along with supportive treatment measures (17). Although various medications have been tested for efficacy, spiramycin is frequently administered to manage maternal infection and inhibit transmission to the fetus (18). Spiramycin, however, may be limited in its ability to cross the placental barrier, potentially reducing its effectiveness in treating the fetus (9). Conversely, the scarcity of this drug in certain countries, due to economic factors and sanctions, has necessitated the exploration of alternative antimicrobial agents such as clindamycin.

Today, clindamycin is widely used as an alternative drug, either alone or in combination with pyrimethamine, in the treatment of toxoplasmosis (19). Several studies conducted in laboratory conditions have investigated the inhibitory effects of clindamycin on *T. gondii*, demonstrating the drug's ability to interfere with the growth and reproduction of the parasite. Additionally, clindamycin in combination with pyrimethamine has been successfully used for the initial and maintenance treatment of toxoplasmic encephalitis in immunocompromised patients and appears to be an effective alternative for those intolerant or unresponsive to standard treatment (20). Clindamycin can cross the placenta and reach umbilical cord serum levels approximately half of those of the mother (11). Evidence strongly supports the safe use of clindamycin during pregnancy, and to date, no known teratogenic effects have been associated with topical or oral clindamycin (21).

Clindamycin has demonstrated encouraging outcomes in animal models, particularly in mice, by diminishing *T. gondii* parasite levels and alleviating toxoplasmosis symptoms. However, data on its dosage for treating congenital infections are scarce (22). A systematic review and meta-analysis indicate that clindamycin used before 22 completed weeks of gestation in women with objective evidence of abnormal genital tract flora can significantly reduce the rate of late miscarriage and early preterm birth (23).

In this study, we assessed clindamycin’s effect on reducing the vertical transmission rate of toxoplasmosis caused by the PRU strain (Type II) in mice. 

In the present study, treatments with clindamycin and spiramycin significantly affected the model of congenital infection when compared to the untreated control group. In cases of infection by 20 cysts from the PRU strain, both drugs significantly improved protection and reduced the remaining infection. Clindamycin achieved a reduction rate of 60.82% in brain samples and 80.95% in eye samples, while spiramycin showed a reduction of 90.72% and 90.48% in these tissues, respectively. However, the difference in eye cyst numbers between the clindamycin and spiramycin-treated groups (positive control) was not significant (P = 0.41). Additionally, clindamycin has demonstrated promising outcomes in animal models, particularly in mice, by lowering parasite burden and alleviating the severity of toxoplasmosis (24). In research by AL-Akash et al., the combined effects of malarone and clindamycin were assessed in a congenital toxoplasmosis model. The synergistic treatment resulted in a 100% survival rate, a decrease in brain tissue cysts, and a reported cure rate of 100% (25).

Quantitative PCR assessment revealed a marked reduction in *T. gondii* parasite load within the brain, eye, and placenta tissues of mice treated with spiramycin and clindamycin compared to untreated controls, indicating the effectiveness of these drugs. This reduction was statistically significant (P < 0.05). Notably, a significant statistical difference was also observed between clindamycin when compared to the positive control group (spiramycin) (P < 0.05).

In this study, the notable decrease in parasite burden observed in the subgroups receiving clindamycin and spiramycin aligns with the results reported by Araujo and Remington. Their research demonstrated that clindamycin is effective in treating both acute and chronic toxoplasmosis caused by the RH strain in mice. Additionally, it highlighted clindamycin’s therapeutic potential to prevent the congenital transmission of the parasite during the acute phase of infection. According to the results of this study, clindamycin not only safeguarded mice against the fatal effects of acute infection but also eradicated the pathogen from host tissues, notably the spleen, liver, and blood, during chronic (latent) infection. Furthermore, clindamycin was successful in preventing the vertical transmission of the infection during acute maternal infection (26).

In our study, we analyzed the histopathological alterations in the brain, eye, and placenta tissues across the treated cohorts, revealing notable significant differences when compared to the control group. Administration of clindamycin, like spiramycin, markedly mitigated histopathological alterations when compared to the control group that received no treatment. The notable reduction in inflammatory changes seen in the subgroups treated with both spiramycin and clindamycin aligns with the findings presented in the 2021 study by AL-Akash et al. (25). In the research carried out by Tabbara et al. in 1974, histopathological analysis indicated that the regular architecture of the retina remained intact in 75% of the eyes that received clindamycin treatment, with only minor to moderate inflammation observed (27). Additionally, these results align with the outcomes presented by Allam et al., which demonstrated that treatment with both spiramycin and its nano-formulated variant could alleviate the pathological impact on the liver, spleen, and brain in mice infected with the RH strain of Toxoplasma (28).

Inflammatory cytokines are pivotal in disease progression and may contribute to tissue damage through lipid peroxidation. Several studies have shown that TNF-α peaks during acute infection, implicating its role in the pathogenesis of acute toxoplasmosis (29, 30). Our study observed a reduction in TNF-α levels following clindamycin and spiramycin treatment, signifying an enhanced cellular immune response. IL-10 is crucial in maintaining the equilibrium between protective immunity and the development of immune pathology. It often exhibits biological activities that counteract those prompted by TNF-α, and it has been observed to inhibit the expression of numerous genes and functions activated by TNF-α (31). In the current study, the elevated IL-10 levels observed in groups infected and treated with clindamycin or spiramycin could contribute to the reduced production of TNF-α (32). Research generally indicates that clindamycin can suppress the growth and reproduction of *T. gondii*, highlighting its potential use in fighting the parasite. Clinical studies suggest that clindamycin’s effectiveness against toxoplasmosis may be similar to its antibacterial action, which involves targeting the ribosomes within the parasite’s cytoplasm and mitochondria (33-35).

Although spiramycin is typically the preferred initial treatment for congenital toxoplasmosis, clindamycin can also serve as a beneficial alternative or supplementary therapy in situations where there is resistance, intolerance, or limited access to primary medications.

### 5.1. Conclusions

This research offers significant findings on the effectiveness of clindamycin in preventing the mother-to-child transmission of *T. gondii*, especially in cases of congenital toxoplasmosis. Although complete eradication of the parasites may not be achieved, the evidence suggests that clindamycin could be an effective component of a broader treatment plan. The decision to use spiramycin or clindamycin in the treatment of congenital toxoplasmosis depends on several factors, including the severity of the infection, gestational age, the patient's medical history, and the availability of specific drugs. Continued investigation into therapy optimization, drug delivery targeting, toxicity reduction, treatment outcome enhancement, and overcoming the barriers of drug penetration into the placenta and central nervous system through drug nanofabrication could advance our knowledge and improve the treatment of congenital toxoplasmosis.

## Data Availability

All data is provided in the manuscript and in additional files. All data are available on request.
